# Phenotypic Evidence of Emerging Ivermectin Resistance in *Onchocerca volvulus*

**DOI:** 10.1371/journal.pntd.0000998

**Published:** 2011-03-29

**Authors:** Mike Y. Osei-Atweneboana, Kwablah Awadzi, Simon K. Attah, Daniel A. Boakye, John O. Gyapong, Roger K. Prichard

**Affiliations:** 1 Institute of Parasitology, McGill University, Montreal, Canada; 2 Onchocerciasis Chemotherapy Research Centre, Hohoe, Ghana; 3 Noguchi Memorial Institute for Medical Research, University of Ghana, Accra, Ghana; 4 Health Research Unit, Ghana Health Service, Accra, Ghana; 5 Council for Scientific and Industrial Research, Accra, Ghana; 6 Ochocerciasis Control Programme of Ghana, Accra, Ghana; New York Blood Center, United States of America

## Abstract

**Background:**

Ivermectin (IVM) has been used in Ghana for over two decades for onchocerciasis control. In recent years there have been reports of persistent microfilaridermias despite multiple treatments. This has necessitated a reexamination of its microfilaricidal and suppressive effects on reproduction in the adult female *Onchocerca volvulus*. In an initial study, we demonstrated the continued potent microfilaricidal effect of IVM. However, we also found communities in which the skin microfilarial repopulation rates at days 90 and 180 were much higher than expected. In this follow up study we have investigated the reproductive response of female worms to multiple treatments with IVM.

**Methods and Findings:**

The parasitological responses to IVM in two hundred and sixty-eight microfilaridermic subjects from nine communities that had received 10 to 19 annual doses of IVM treatment and one pre-study IVM-naïve community were followed. Skin snips were taken 364 days after the initial IVM treatment during the study to determine the microfilaria (mf) recovery rate. Nodules were excised and skin snips taken 90 days following a second study IVM treatment. Nodule and worm density and the reproductive status of female worms were determined. On the basis of skin mf repopulation and skin mf recovery rates we defined three categories of response—good, intermediate and poor—and also determined that approximately 25% of subjects in the study carried adult female worms that responded suboptimally to IVM. Stratification of the female worms by morphological age and microfilarial content showed that almost 90% of the worms were older or middle aged and that most of the mf were produced by the middle aged and older worms previously exposed to multiple treatments with little contribution from young worms derived from ongoing transmission.

**Conclusions:**

The results confirm that in some communities adult female worms were non-responsive or resistant to the anti-fecundity effects of multiple treatments with IVM. A scheme of the varied responses of the adult female worm to multiple treatments is proposed.

## Author Summary

Onchocerciasis, commonly known as river blindness, is caused by the filarial nematode *Onchocerca volvulus* and is transmitted by a blackfly vector. Over 37 million people are thought to be infected, with over 90 million at risk. Infection predominantly occurs in sub-Saharan Africa. Foci also exist in the Arabian Peninsula and Central and South America. Ivermectin, the sole pharmaceutical available for mass chemotherapy, has been used on a community basis for annual or semi-annual treatment since 1987. Multiple treatments with ivermectin kill the microfilariae that are responsible for the pathology of onchocerciasis. More importantly, ivermectin suppresses the reproductive activity of the adult female worms, thus delaying or preventing the repopulation of the skin with new microfilariae and thereby reducing transmission. This study extends earlier reports of sub-optimal responses to ivermectin by examining repopulation levels of microfilaria one year after treatment, worm burdens per nodule, the age structure of adult female worms recovered from nodules, and the reproductive status of adult female worms 90 days after ivermectin treatment. In some communities which have shown a pattern of sub-optimal response to treatment, the data is consistent with an emergence of ivermectin non response or resistance manifested by a loss of the effect of ivermectin on the suppression of parasite reproduction.

## Introduction

Ivermectin (Mectizan) has been in operational use for the control of human onchocerciasis in the former Onchocerciasis Control Programme in West Africa (OCP) areas since 1987 and is still the drug of choice for the African Programme for Onchocerciasis Control (APOC) and the Onchocerciasis Elimination Programme for the Americas (OEPA). A single dose of ivermectin rapidly kills skin microfilariae (mf) and inhibits microfilarial release by adult female worms, possibly because of paralysis of the lower uterus [1]. These effects result in a rapid decline in skin microfilarial counts, an accumulation of degenerating intra-uterine microfilariae and a long-lasting suppression of *Onchocerca volvulus* microfilaridermias [2]. However, the inhibition of mf release is reversible by six months resulting in the reappearance of microfilariae in the skin (repopulation) and after a further six months a restitution of a proportion of the initial load (recovery) [2]. A proportion of ivermectin exposed female worms do not resume reproductive activity even after one year [1] and this can persist for up to 18 months after treatment [3]. The prolonged microfilarial suppressant effect of ivermectin has beneficial effects on morbidity and on parasite transmission.

Several studies [1], [4], [5], [6], [7], [8] have demonstrated that multiple treatments with ivermectin have marked suppressive effects on embryogenesis consistent with some cumulative effect of ivermectin treatments on microfilarial production. Quantitative estimates have ranged from an irreversible decline in microfilarial production of ∼30% after five annual treatments [9], a reduction in the productivity index of 90% or more after 10 six-monthly doses over 6 years [10], to arrest of development at the single cell stage after four or five six-monthly doses [11]. Recently, the cumulative effect of ivermectin on microfilarial production by *O. volvulus* has been questioned [12] based on mathematical modeling, with a number of necessary assumptions, of data generated in three Guatemalan villages that were subjected to five 6-monthly treatments. The conclusions from this study are, however, not consistent with data generated previously in Africa. Despite the varying dosage schemes, methodologies, criteria of assessment and interpretation of results that have been used, it is evident that the presence of a significant proportion of adult female worms producing viable microfilariae 3 to 6 months after treatment, following multiple doses of IVM, is inconsistent with a normal response. A sub-optimal response is manifested as higher than expected skin microfilaria counts at various time points post treatment. Awadzi and others [13] have proposed that for effective response of *O. volvulus* to IVM, the mf load in a patient one year after the last IVM treatment should not be >10 mf/snip following nine or more years of annual treatments.

Despite previous African experience there are current reports of persistent, moderately high microfilaridermias after repeated ivermectin treatments [13], [14], [15]. These reports suggest that a population of adult female worms may have been selected that responds poorly to ivermectin. The development of resistance to ivermectin and other macrocyclic lactone anthelmintics has become widespread and is increasing in nematode parasites of sheep, goats and cattle, including *Haemonchus contortus* and *Cooperia onchophora* [16], [17], [18], [19]. With the distribution of more than 400 million doses of ivermectin as the only drug for mass onchocerciasis control in Africa [20] and with some communities having received more than 19 annual doses, it would not be surprising that the genetic selection [21], [22], [23], [24] observed in *O. volvulus* with IVM treatment was indicative of the development of ivermectin resistance in this parasite.

We have previously reported moderately high early repopulation of skin with microfilariae by some populations of adult *O. volvulus* [15]. Following this report, we have reassessed the differences between *O. volvulus* populations showing poor responses and those showing good responses to ivermectin using various indicators of skin microfilarial repopulation, nodule and worm viability, adult worm densities, and female worm fertility in the original 10 endemic communities.

## Methods

### Study Design

**Figure 1:**
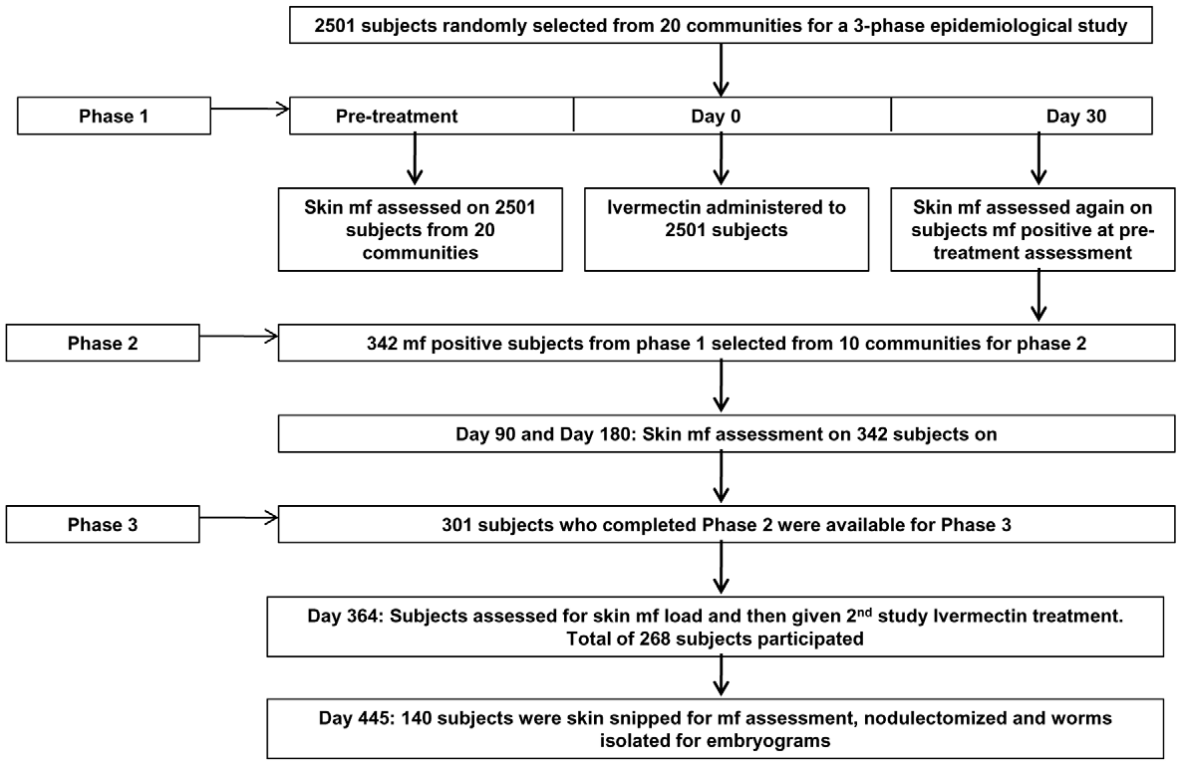
Study design. Design for the entire study, showing time points used for examination of various parameters and the study populations at each phase of the study.

This was a randomized, open 21 month longitudinal study involving two annual ivermectin treatments, serial skin snipping and nodulectomies in the communities under a study that was carried out between October, 2004 and June, 2006. Skin microfilarial profiles were assessed in onchocerciasis affected patients at various time-points pre- and post-treatment to determine skin mf repopulation and, at one year post treatment, the microfilaria recovery rates. At the end of the study (90 days after a second annual treatment during the study), nodules containing adult *Onchocerca volvulus* were surgically removed and digested to extract adult worms. Embryogrammes were constructed to assess the reproductive status of the adult female worms. A summary of the study design and conduct is shown in Figure 1.

### Study Population and Area, Subject Eligibility and Selection

The study was carried out in 10 onchocerciasis endemic communities located in three Districts in Ghana; Kintampo and Atebubu Districts in the Brong-Ahafo Region, and Gonja East District in the Northern Region. The criteria for selection of the communities have been detailed previously [15] and for the 9 communities that had received multiple treatments include: good written documentation on annual community treatment coverage, good community and individual treatment history, at least one survey over the previous 6 yrs (prevalence, community microfilarial load or both), average treatment coverage ≥50% in the previous 5 years, uninterrupted annual treatment over the previous 6 years and ivermectin treatment within the previous 10–12 months. Additionally, communities had to be accessible by road throughout the entire study period, especially during the period for nodulectomies. One community was ivermectin-naïve prestudy.

Ethical approval for the study was obtained from the Institutional Review Board of Noguchi Memorial Institute for Medical Research, Ghana and the Ethical Review Board of McGill University, Canada. The informed consent procedure involved a durbar (meeting with the chief, elders and the entire community) with the study population at which the study design, investigative procedures and the risks and benefits of participation were outlined by the principal investigator in English and in the local language through an interpreter. Unlimited time was allowed for questions and explanations. After the durbar individuals who were interested in participation met with the investigator and interpreter individually and the contents of the consent form were explained in detail. After further questions and explanations, each subject signed or thumb-printed an informed consent form that testified to the fact that they had been told the details of the study, any questions they had asked had been answered to their satisfaction and that they freely consented to participate in the study.

### Investigative Procedures

Subjects were aged between 18–65 years, had lived in the communities for at least ten years and, with the exception of the prestudy ivermectin-naïve, had received between 10 and 19 annual doses of IVM confirmed by interviews of participants and community ivermectin distributors, and examination of written treatment records, and had been skin snip positive for *O. volvulus* at the beginning of the study.

All subjects were examined in detail for Onchocerca nodules and the locations of the nodules were recorded on anatomical diagrams. One year after the last ivermectin treatment (364 days), one skin snip was taken from each iliac crest using a 2 mm Holth-type corneo-scleral punch, by the same member of the study team, who was experienced with conducting skin snips, to maintain consistency. A second annual ivermectin treatment was then given during the study, as part of Ghana Onchocerciasis Control Program, to all subjects at 150µg/kg body weight. The final skin snips were then taken by the same operator only from subjects who were nodulectomised 90 days after the second IVM treatment. Skin snips were placed in 96-well microtitre plates containing a few drops of physiological saline solution, incubated for 24 hours and microfilariae that had emerged were counted using a dissecting microscope. The average of the microfilarial counts from the two sites was taken as the intensity of infection for each subject expressed as microfilariae/snip.

Nodulectomies were performed 90 days following the second ivermectin treatment, i.e. day 455 after the first study treatment. Using local anesthesia, all palpable Onchocerca nodules were aseptically excised from 140 patients from the 10 endemic communities. Nodules were stored in liquid nitrogen until ready for digestion. Nodules were dressed free of extraneous tissue and placed in 50 ml tubes containing 10 ml of 0.5% collagenase (in sterile medium 199 solution) for digestion at 37°C in a shaking water bath for 10–24 hours. Adult worms were harvested after washing with sterile normal saline solution, and under a dissecting microscope, the viability and morphological age of worms at the time of nodulectomy were scored and intact/viable female worms prepared for embryogramme analysis. Worms were classified as alive prior to nodulectomy based on intact internal morphology, motility of the worms, and the condition of the uterine musculature Broken or ruptured worms were not examined for embryogrammes. The age of the worms was estimated based on the morphology, including the color and size of the female worms, the prominence of cuticular ridges and the degree of inclusions [10], [11], [25]. In addition to the above criteria, small and transparent worms were scored as young, opaque and yellowish as middle aged, and large and brown as older [25], [26]. Each intact female worm was cut into small pieces, two millilitres of fresh sterile medium 199 was added and worms homogenized using a toughened glass test tube mortar and pestle. By turning the pestle gently, the embryonic stages were squeezed out of the pieces of worms and embryogrammes constructed [27]. The homogenate was transferred to a Fuchs-Rosenthal counting chamber and all embryonic stages assessed as described previously [26], [27]. Quantitative assessment of normal and abnormal forms of each embryonic stage up to stretched microfilariae was done to determine the reproductive status and microfilarial content of each individual female worm [28].

### Data Management and Statistical Analysis

After data verification, the mean microfilarial density (mf/snip) at each time point and the density as a percentage of the initial count were determined for each community. The number of subjects in each community whose skin mf densities were greater than, the same as, or less than the initial density, or who were skin snip negative, was defined, as well as the number of subjects with greater than 10 mf/snip at day 364. Based on skin mf repopulation (early reappearance of microfilariae in the skin at day 90 and/or day 180 post-IVM treatment determined in the initial study), the mf recovery rate (skin mf density, at day 364, as a percentage of the initial count) and the distribution of the number of subjects with >10 mf/snip at day 364, communities were reclassified into four response categories (good, intermediate, poor, and the pre study ivermectin-naïve that was used as the comparator group). The intermediate response category shared some of the response characteristics of the poor response category, but not all of the characteristics. The nodule data for the communities in each response category were pooled separately and these pooled data sets were used for the analysis of the findings in the embryogrammes.

Comparisons of microfilarial densities, mf recovery at day 364 and mf repopulation at day 455 (90 days after the second study IVM treatment) between the 10 communities were carried out on log transformed data using the Kruskal-Wallis non-parametric test and pair-wise comparisons by the non-parametric Mann-Whitney test. Comparison between the 10 communities in terms of nodule characteristics and worm density and between the IVM response categories regarding prevalence, reproductive activity of adult female worms and embryogramme analysis were carried out using the Chi-square test or Fisher's exact test where appropriate. Differences were considered as significant at p< 0.05.

## Results

Two hundred and sixty eight of the 301 microfilaridermic subjects from the 10 communities who had fully participated in the previous phase of the study [15] were available and agreed to continue in the follow up study. The results for pretreatment and microfilaria assessment up to day 180 have already been reported [15]. In that report, the microfilariae in all communities responded readily to ivermectin with reductions of 99.3 to 100% being achieved at day 30. However, we found that while in five of the nine communities that had received multiple doses of ivermectin (Baaya, Beposo, Senyase, Hiampe, and Asubende) and in the ivermectin naïve community (Begbomdo) the repopulation rate was as expected and the response was classified as good, in four multi dosed communities (Kyingakrom, New Longoro, Jagbenbendo and Wiae) individual hosts had higher than expected skin mf counts and the response was classified as poor.

The skin microfilarial indicators that were used in this study to indirectly assess the reproductive capacity of the adult female worms were the microfilaria recovery rate (% of pre-treatment level at day 364 post IVM treatment), the distribution pattern of mf recovery rates of individuals in each community, the number of subjects with >10 mf/s at day 364 across the communities and the mf repopulation 90 days after the second IVM treatment during the study (day 455) in the 140 subjects that participated in the nodulectomies.

The mf recovery rate for the various communities is summarized in Table 1. This shows that the microfilaria recovery rate was less than 80% in four of the five multidosed communities previously classified as good responders; the exception was Asubende which had a recovery rate of 103.8%. Three of the four communities previously classified as poor responders, had microfilaria recovery rates of more than 110%; the exception was Wiae with 99.1%.

**Table 1:** Densities of *O. volvulus* microfilaria before, and at 364 days after treatment, in each community.

Community	No. of treatments	No. of subjects examined on day -7 N = 268	Microfilaria/snip at		
			Day -7	Day 364 post-treatment	
				Density	% of day -7 * (recovery rate)
Senyase	18	10	1.69	1.21	71.6
Beposo	18	10	2.24	1.62	72.3
Hiampe	17	17	3.03	2.37	78.2
Baaya	18	20	1.38	0.91	65.9
Asubende	19	12	2.40	2.49	103.8†
Wiae	10	22	3.42	3.39	99.1†
Kyingakrom	17	27	6.40	7.41	115.8‡
New Longoro	17	62	5.73	6.32	110.3‡
Jagbenbendo	12	51	8.17	9.22	112.9‡
Begbemdo¶	1	37	30.74	12.89	41.9§

Geometric mean densities (mf/skin snip) in the ten Ghanaian communities participating in the study.

*Data for % of day -7 are based on the subjects that participated at all sampling times up to day 364.

**†:** p<0.05 when compared to the good responders.

**‡:** p< 0.01 when compared with good responders or prestudy IVM-naïve.

**§:** p<0.05 when compared to good responders. Good responders  =  Senyase, Beposo, Hiampe, Baaya. Intermediate responders  =  Wiae, Asubende. Poor responders  =  Kyingakrom, New Longoro and Jagbenbendo.

**¶:** Ivermectin naïve at study start.

We compared mf skin recovery rate at day 364 post IVM treatment (Table 1) between all the 10 communities. Pair-wise comparisons showed that at day 364 there were no differences in mf skin recovery between the four multi-dosed communities (Senyase, Beposo, Hiampe, Baaya). However, Begbomdo, the pre-study IVM naïve community, had significantly lower (p < 0.05) mf recovery rate than the four communities. These five communities had significantly lower mf recovery than Kyingakrom, Jagbenbendo and New Longoro (p < 0.01) and Wiae and Asubende (p < 0.05).

The distribution pattern of mf recovery rates of individuals in the communities is summarized in Table 2. The good ivermectin response communities had 0–6% of subjects with an mf recovery rate of more than 100% of pretreatment counts compared with 40.3%–51.9% of the subjects in the poor ivermectin response communities. Between these two groups were Asubende (33.3%) and Wiae (27.3%). Additionally, in the good response communities, between 55–80% of the subjects had reductions in microfilarial counts, with 12–20% being amicrofilaridermic one year after treatment, while in the poor response communities only 26–32% of subjects had reductions and very few to zero. When the three variables (proportion of individuals having greater than pretreatment mf densities, same as pre-treatment mf densities and less than pre-treatment densities) were compared together between the 10 communities, Kyingakrom, New Longoro and Jagbenebendo were significantly higher (p<0.01) than five communities (Senyase, Beposo, Baaya, Hiampe and Begbomdo). Also Wiae and Asubende were higher than the four good responding communities and the naïve community, but this was not significant.

**Table 2:** Distribution pattern in each community of microfilarial densities one year after ivermectin treatment.

Community	Total No. of subjects	% (No.) of subjects with day -7 mf density >10 mf/s	% (No.) of subjects with day 364 mf density:				
			> 100% of pre-treatment	The same as pre-treatment (100%)	Less than pre-treatment mf density (0-99%)	Reduced to zero (0%)	> 10 mf/s
Senyase	10	10 (1)	0	20.0 (2)	80.0 (8)	20.0 (2)	0
Beposo	10	10 (1)	0	20.0 (2)	80.0 (8)	20.0 (2)	0
Hiampe	17	17.6 (3)	5.9 (1)	23.5 (4)	70.6 (12)	11.8 (2)	11.8 (2)
Baaya	20	5 (1)	5.0 (1)	35.0 (7)	55.0 (11)	20.0 (4)	5(1)
Asubende	12	16.7 (2)	33.3 (4) n/s	16.7 (2)	50.0 (6)	16.7 (2)	16.7 (2)
Wiae	22	22.7 (5)^3^	27.3 (6) n/s	27.3 (6)	45.5 (10)	13.6 (3)	22.7 (5)^4^ n/s
Kyingakrom	27	40.7 (11)^6^	51.9 (14) §	22.2 (6)	25.9 (7)	3.7 (1)	48.1 (13)^10^ §
New Longoro	62	25.8 (16)^7^	40.3 (25) §	24.2 (15)	32.3 (20)	3.2 (2)	30.6 (19)^10^ §
Jagbenbendo	51	37.3 (19)^9^	43.1 (22) §	25.5 (13)	31.4 (16)	3.9 (2)	43.1 (22)^14^ §
Begbomdo¶	37	81.1 (30)^29^	2.7 (1)	0	97.3 (36)	2.7 (1)	70.3 (26)^13^ §

Three variables, proportion of individuals having (a) greater than pretreatment mf density, (b) same as pre-treatment mf density, and (c) less than pre-treatment mf density, were compared in the 10 communities. Kyingakrom, New Longoro and Jagbenbendo (poor responding communities) were significantly higher (§ p<0.01) than the five communities (Senyase, Beposo, Baaya, Hiampe and Begbomdo) that responded well to IVM treatment. The three poor responding communities were also higher than Wiae and Asubende (intermediate response communities) but the differences were not significant.

**¶:** Ivermectin naïve at start of study. n/s  =  not significantly different from good responders. The superscript in the table e.g., (5)^10^, represents the number of subjects that had >20 mf/s.

A further comparison examined the number of subjects with >10 mf/s at day 364 across the communities (Table 2). There were 3/57 (5.3%) for Baaya, Beposo, Senyase, Hiampe, the good responding communities 7/34 (20.7%) for Asubende and Wiae and 54/140 (38.6%) for Kyingakrom, New Longoro and Jagbenebendo, the poor response communities. The pre-study IVM-naïve community, which at day 364 post the first treatment during the study had only received a single IVM treatment, had 26/37 (70.3%) subjects with >10 mf/s. The difference between the good response and Asubende-Wiae communities was not significant (p = 0.197). However the proportion of subjects with >10 mf/s in the good response communities was significantly lower than in the poor response and the IVM-naive communities (p<0.0001). Asubende and Wiae communities were lower than the poor response (p<0.05) and pre-study IVM-naïve (p<0.0001) communities. Across all multi-dosed communities, 27.7% (64/231) of the subjects had >10 mf/s at day 364 and, given the IVM treatment history, would satisfy the field definition of suboptimal responders [13]. Though this does not differ significantly from 25.5% (59/231) of the multi-dosed subjects who had >10 mf/s at the beginning of the study (Table 2), the good response communities had a significant reduction in the proportion of subjects who had >10 mf/s from 10.5% (6/57) at day -7 to 5.3% (3/57) at day 364. For the Asubende-Wiae communities, there were no changes, but the poor response communities showed a slight increase in the proportion of subjects who had >10 mf/s, from 32.9% (46/140) at day -7 to 38.6% (54/140) at day 364 and those who had >20 mf/s from 15.7% (22/140) to 24.3% (34/140).

The determination of the skin mf repopulation 90 days after the second IVM treatment during the study (day 455) on the 140 subjects that participated in the nodulectomies is summarized in Table 3. The overall result was similar to that reported for the day 90 mf repopulation observed in the same group of individuals from the ten communities in the first phase of the study (15). We observed no skin mf repopulation in the four good response communities but a small repopulation (2.9% of pretreatment count) in the prestudy naïve community. In each of the three poor response communities the level of skin mf repopulation was greater after the second study treatment (day 455) than after the first study treatment (day 90), but the differences were not significant.

**Table 3:** Densities and microfilaria repopulation rates after first (day 90) and second ivermectin treatments (day 455).

Community	No. of treatments at study end	No. of subjects nodulectomized and skin snipped day 455N = 140	Microfilaria*/snip at			
			day -7	Day 90mf/s (% day -7)	Day 364	Day 455mf/s (% day 364)
Senyase	18	8	1.9	0 (0)	1.3	0 (0)
Beposo	18	5	2.4	0 (0)	1.7	0 (0)
Hiampe	17	7	3.3	0.06 (0.02)	2.2	0 (0)
Baaya	18	6	1.9	0 (0)	1.2	0 (0)
Asubende	19	5	3.2	0 (0)	3.38	0.08 (0.02)
Wiae	10	10	4.1	0.28 (6.8)	4.0	0.2 (5.0)
Kyingakrom	17	20	7.9	1.7 (21.5) †	9.1	2.1 (23.1) †
New Longoro	17	20	7.2	0.63 (8.8) †	8.1	0.99 (12.2) †
Jagbenbendo	12	39	8.2	0.94 (11.5) †	9.16	1.1 (12.0) †
Begbemdo ¶	2	20	44.2	1.4 (3.2)	17.3	0.5 (2.9)

Geometric mean densities (mf/skin snip) of *O. volvulus* microfilaria and repopulation rates observed at day 90 following the first study IVM treatment (day 90) and second IVM treatment (day 455) in the 10 onchocerciasis endemic communities studied in Ghana.

*All data are based only on the subjects that were nodulectomized. At days 90 after the first study treatment and day 90 after the second study IVM treatment (i.e., day 455), there were no differences in skin mf repopulation (mf count as a % of pre-treatment) between five communities (Senyase, Beposo, Hiampe, Baaya and Begbomdo – good response or naive communities). However, three communities (New Longoro, Jagbenbendo and Kyingakrom) had significantly higher († p< 0.05) skin mf repopulation rates (%) than the good response communities.

¶ Previously naïve had received two study treatments.

When the results of the indices of skin repopulation by mf are taken together they show a gradation of response to multiple doses of ivermectin that permits the re-classification of the responses as good (Senyase, Beposo, Hiampe and Baaya), intermediate (Asubende and Wiae), and poor (Kyingakrom, New Longoro and Jagbenbendo). The response to ivermectin in the previously treatment naïve community (Begbomdo) was also good based on mf repopulation levels one year after treatment as a proportion of pretreatment mf levels.

Nodulectomies were carried out on a total of 140 patients from the ten communities who agreed to participate in the procedure. Two hundred and ninety three nodules were removed, giving an average of 2.1 nodules per subject. In order to better quantify the nodule characteristics and the embryogramme findings, the data were pooled separately for communities labeled as good, poor and intermediate responders on the basis of skin microfilaria repopulation and recovery rates. The nodule characteristics and distribution of adult worm populations are shown in Table 4. Overall, 100 out of 140 subjects (71.4%) had nodules containing at least one viable worm. The features compared were the number of subjects with nodules containing viable worms, number of nodules with viable worms and the average number of male and female worms per nodule. The prestudy ivermectin naive (Begbomdo), and the poor response communities were significantly higher (p < 0.0003) than the good response communities in terms of nodules with viable worms and the average number of male and female worms per nodule as well as the number of subjects with nodules containing viable worms (p < 0.01). The intermediate response communities were significantly higher (p <0.002) than the good response communities only in the average number of male and female worms per nodule.

**Table 4:** *O. volvulus* nodule characteristics and worm densities in the IVM response/treatment categories.

Response/treatmentcategories	IVM treatment history	No. of subjects nodulectomised	No. of nodules removed	No. (%) of subjects with nodules containing viable worms	No. (%) of nodules with viable worms	No. of male worms	No. of female worms	Average No. of females per nodule	Average No. of males per nodule
Begbomdo¶	2	20	46	19 (95.0)†	42 (91.3)§	68	104	2.26§	1.48§
Good	18–19	26	51	7 (26.9)	12 (23.5)	8	18	0.35	0.16
Intermediate	11–20	15	29	10 (66.6)	16 (55.2)	19	36	1.24*	0.66*
Poor	13–18	79	167	64 (81.0)†	130 (77.8)§	180	258	1.54§	1.08§
Total		140	293	100	200	275	416	1.42	0.94

The number of subjects with nodules containing viable worms, number of nodules with viable worms, and average number of males and females per nodule were compared between the different IVM response categories (see Table 2 for allocation of communities to response categories) and the IVM naïve community. Begbomdo and poor response communities were significantly higher (§ p < 0.0003) than the good response communities in terms of number of nodules with viable worms and average number of male and female worms per nodule, as well as the number of subjects containing viable worms († p < 0.01). The intermediate response communities were significantly higher (* p < 0.002) than the good response communities in the average number of male and female worms per nodule.

¶ Ivermectin naïve at start of study.

The age structure and the state of reproductive activity of adult female worm populations in endemic communities give important information on parasite transmission and the contribution of each parasite age group to the repopulation of the skin with microfilariae after IVM treatment. The adult female worms were grouped into older, middle aged and young worms as described previously and are presented by response category in Tables 5A and B. In all three categories of response to multiple doses of ivermectin at least half of the worms were older (52–78%) and approximately one quarter were middle aged (22–30%). The proportion of young worms was only 5.1% and 8.1% in the poor and intermediate response categories respectively and there were no young worms found in the good response category. This proportional distribution by age (52–78% for the older worms and 22–30% for the middle aged worms) was similar in all response categories except the prestudy ivermectin naïve that had a significantly higher proportion of young worms (21.2%; p< 0.002) than all multidosed response categories (0–8%). When all the worms were pooled and compared by age group there were significantly more older (65.4%) than middle aged (25.5%; p = 0.002) and young worms (9.1%; p<0.0001), and more middle aged worms than young worms (p<0.0001) (Table 5).

**Table 5:** Age structure of all female *O. volvulus* worms sampled in IVM response/treatment categories.

Community response/treatment category	No. of years of IVM treatment	% (No.)older female worms	% (No.)middle agedfemale worms	% (No.)young female worms	Total number of female worms
Begbomdo ¶	2	51.9 (54)	26.9 (28)	21.2 (22)§	104
Good	18–19	77.8 (14)	22.2 (4)	0	18
Intermediate	11–20	62.2 (23)	29.7 (11)	8.1 (3)	37
Poor	13–18	70.4 (181)	24.5 (63)	5.1 (13)	257
Total	-	65.4 (272)	25.5 (106)	38 (9.1)	416

The IVM naïve community had significantly higher numbers of young worms (§ p< 0.002) than each of the IVM community response categories. The worm age distribution was not significantly different between the good, intermediate and poor response categories.

¶ Begbomdo was IVM naïve at the start of the study.

An examination of the live stretched mf production by the female worms showed clear differences between the 3 response groups. In the good response category, none of the worms examined was producing mf. In the intermediate response category 5% of the old worms and 10% of the middle aged worms were producing mf but none of the young worms. In the poor response category 15.1% of the old worms, 22.2% of the middle aged worms and only 0.8% of the young worms (1 worm) were producing mf. Overall, 12.2% of the older worms, 17.2% of the middle aged worms and only 1.1% of the young worms were producing mf (Table 6). The differences between the older and middle aged worms combined and the young worms were highly significant (p<0.0001) but not that between the older and middle aged worms.

**Table 6:** Prevalence and reproductive activity of young, middle-aged and older female worms in the response/treatment categories.

Response/treatment category	No. female worms Embryogrammed	No. (%) olderfemale worms		No. (%) middle agedfemale worms		No. (%) young femaleWorms	
		Total	Producinglive stretched mf	Total	Producinglive stretched mf	Total	Producinglive stretched mf
Begbomdo¶	20	9 (45.0)	2 (10)	6 (30.0)	1 (5)	5 (25.0)	1 (5)
Good	14	10 (71.4)	0	4 (28.6)	0	0	0
Intermediate	20	10 (50.0)	1 (5)	7 (35.0)	2 (10)	3 (15.0)	0
Poor	126	66 (52.4)	19 (15.1)	48 (38.1)	28 (22.2)†	12 (9.5)	1 (0.8)
Total	180	95 (52.8)	22 (12.2)	65 (36.7)	31 (17.2) ‡	20 (11.1)	2 (1.1)

In each IVM response category, the proportion of female worms in each age group, producing intra-uterine live stretched mf, were compared. In the poor response communities, the middle aged worms had a significantly higher († p<0.05) proportion of female worms producing intra-uterine live stretched mf than the older and young worms. Pooling all response groups together, the middle aged worms had significantly higher (‡ p<0.03) proportions of female worms producing intra-uterine live stretched mf than the older and young worms.

¶ Begbomdo was naïve at the start of the study and had received two study treatments by time of nodulectomy.

In order to more precisely quantify the contribution of the various age groups of adult worms to the skin microfilariae, the various age categories were pooled across all response groups and the mean production of stretched microfilariae calculated. The results are summarized in Table 7. The mean of the production of total (live and degenerate) stretched microfilariae/female worm was significantly higher in the middle aged worms as compared to the young worms (p = 0.039), but the older worms were not significantly different from the young worms (p = 0.21). The middle aged worm production of live stretched microfilariae/female worm was higher than those of the older (p = 0.042) and the young worms (p = 0.033). There was no significant difference between the numbers of live stretched microfilariae in the young worms compared with the older worms.

**Table 7:** Reproductive status of female worms from multiply treated subjects, 90 days after ivermectin.

Female worm age group	No. wormsembryogrammed	Stretched microfilariae				
		Total stretched microfilaria			Live stretched mf (active)	
		Mean/ worm	Range	% degenerate	Mean/worm	Range
Older aged	95	2829.0	0–14080	84.5%	437	0–6400
Middle aged	65	3141.3‡	0–13440	70.4%	930.6†	0–8960
Young	20	1874.2	0–12800	80.5%	365.7	0–5120

Reproductive status of multiple IVM treated female worms 90 days after the second ivermectin treatment during the study; showing mean microfilarial numbers of total and active (live) stretched microfilariae by age of female worm. The middle aged worms had a significantly higher (‡ p≤0.05) number of microfilariae in the uterus than the young worms. Also the middle aged worms had a significantly higher († p≤ 0.05) number of live stretched mf than both young and older worms.

## Discussion

In communities where ivermectin is distributed annually as the sole measure of onchocerciasis control, the interpretation of skin microfilarial counts over time is governed by proof of regular consumption of a minimum number of doses, the microfilaricidal response, the sensitivity of the adult female worms to the suppression of embryo production, the effects of ivermectin on microfilarial release and the confounding effect of new microfilarial production by young worms derived from reinfection from local or imported sources. Comparison is then made with historical data that define microfilaricidal activity (usually determined at day 30), repopulation (determined at days 90 and 180) and recovery (determined at day 364). Supportive evidence is best obtained from embryogrammes examined at defined intervals after drug administration. In our previous study [15] extraordinary measures were taken to ensure that the number of treatments allotted to each member of the study cohort was plausible. The sensitivity of microfilariae in all communities was documented as was the abnormal repopulation rates in some communities. This follow up study sought further evidence of deviations from expected skin mf counts as manifested by mf recovery rates, adult worm status as defined by nodule and worm viability and adult worm densities, and provided quantitative estimates of adult female worm distribution and microfilarial production for various worm age groups in the original 10 endemic communities. The microfilaria recovery rates are good indicators for the responses of *O. volvulus* to repeated rounds of IVM treatment because a premise of the control programs is that skin mf counts will progressively decrease year after year with repeated ivermectin treatment when coverage is good. Thus unexpected recovery rates act as a stimulus to the investigation of the cause of persistent microfilaidermia.

Apart from the community recovery rate, the pattern of recovery in individuals in each community and the number of subjects with >10 mf/s at day 364 across the communities were examined. Our results showed that day 364 post treatment skin microfilaria levels of good response communities, including the IVM-naïve community, were below pre-treatment levels, while the poor response communities were above pre-treatment counts, with the intermediate response communities showing mf densities around pretreatment levels.

Studies have shown that irrespective of the initial levels of pre-treatment microfilarial load, repeated annual treatment with IVM for five years or more results in reduction of microfilarial loads to a mean microfilaria density of less than 10 mf/s one year after the fourth or fifth treatment [9], [29], [30]. The classical observation is the study by Alley and others [29] who, using skin snip methodology similar to that employed in this study, consistently followed up more than 260 subjects every year after each treatment for five years. They observed a drastic reduction in the mean microfilaria counts from >90 mf/s to <8 mf/s. Our results for the good response communities are consistent with these reported findings. In contrast, the poor response communities had mean mf counts of >10 mf/s to as high as 16.54 mf/s. Furthermore, considering the distribution of microfilarial loads of individual subjects in each community (Table 2) we observed significantly higher proportions (30–48%) of subjects in poor response communities having >10 mf/s as compared to 16–23% in the intermediate response communities and 0–12% in the good responding communities despite repeated ivermectin treatments over more than 10 years. Awadzi and others [13] defined sub-optimal responders as individuals who still had at least 10 mf/snip after nine or more treatments with ivermectin. It should be noted that even in two of the good response communities we observed that one or two individuals had mf loads slightly above 10 mf/s and we believe that even in the good responding communities there may be some parasites in a few individuals showing a poor response to IVM. The use of the >10 mfs/snip criterion in assessing recovery rates is particularly useful as it is independent of initial mf densities and of percentage change in initial densities. Additional skin snip data obtained 90 days (day 455) after a second dose of IVM treatment during the study, confirmed the earlier observation of rapid skin mf repopulation in the poor response communities.

The skin snip data and their analysis permit the conclusion that a higher than expected return of mf to the skin occurred in some communities. The explanation for this finding is that either the adult female worms have become unresponsive or resistant to the suppressive effects of multiple doses of IVM or that new infections from ongoing transmission could account for the higher than expected rate of repopulation of the skin with microfilariae. This latter possibility was statistically analyzed [31] and the conclusion was reached that the patterns in the communities showing sub-optimal responses could not be explained by new infections. Furthermore, the distinction between the higher than expected microfilarial repopulation rates due to new infections or due to resistance of the adult worms to the effects of ivermectin on reproduction can also be made on the basis of the examination of the age distribution and embryogrammes of the adult worms. On the basis of the age distribution of the adult female worms, the embryogrammes and the statistical analysis [31], the only explanation for the rapid repopulation rates seen in the poor response communities is that individual worms exist in these communities which do not respond as expected to ivermectin.

Repeated IVM treatment has marked effects on nodule numbers, morphology and composition, on adult female worm fertility and a less marked effect on adult worm vitality. Additionally, a reduction in transmission results in a decrease in new infections [3], [5]. Klager and others [11] reported a marked tendency towards smaller nodules and fewer live females per nodule with increasing length of exposure to ivermectin. They also showed that the geometric mean number of worms per nodule and total live female and male worms per nodule were significantly reduced after 10 doses of IVM. For our study, all treated communities had received at least ten years of annual IVM treatment and we expected a similar effect of ivermectin on the viability of both nodules and adult worms. However, our results showed significant differences between the good and poor response communities. In the good response communities, more than 65% of nodules were calcified and contained a mean of 0.35 female and 0.16 male worms per nodule (Table 4). On the other hand the average numbers of male and female adult worms per nodule in the poor response communities were similar to those in the pre-study IVM-naïve community. In view of the fact that there were few young worms which could suggest recent infection, this result suggests a non response or resistance to multiple doses of IVM.

The proportions of older (62–78%) and middle aged worms (22–30%) in all three community response categories were similar. There were only 5% and 8% of young worms in the poor and intermediate response communities, respectively, and none in the good response communities. Additionally, the most reproductively active group was the middle aged worms followed by the older and then the young female worms. We conclude that the major contributors to the skin mf population are not the young adult worms. They must of necessity be the worms that are a few to many years older than the young worms. Since these are the worms that must have been exposed to multiple treatments with IVM in their lifetime (and two IVM treatments during the study itself) they represent non responders or worms that are resistant to the suppressive effects of multiple doses of IVM on mf production. However, the high proportion of degenerate stretched mf *in utero* found in all communities indicates the retention of the ability of IVM to prevent the release of the mf in some worms.

It has been suggested [32], [33], that an alternative explanation for the high skin microfilarial repopulation rates is the occurrence of repeated re-infections due to poor coverage in the study communities and in surrounding communities. However, all of the communities categorized as poor or intermediate responders had good records of treatment coverage. While treatment coverage in some communities in the East Gonja district (where the pre-study treatment naïve community is found) may have been poor, coverage in surrounding communities in the Atebubu and Kintempo districts was generally good (Osei-Atweneboana and Prichard, unpublished). The mechanism underlying the alternate hypothesis must be the recruitment of significant numbers of new adult worms that would account for the skin mf load. Notably, the embryogrammes from the multi-dosed communities showed a dearth of young adult worms at a modest level of reproductive activity. These findings do not support the alternative hypothesis, nor does the analysis of the annual transmission rate that would be required to account for the skin mf repopulation rates observed in the poor responder communities [31]. However, support for the alternate hypothesis can be found in the pre-study IVM-naïve community where coverage was poor and where there were a relatively high proportion of young adult female worms (21%) as compared to 5% in the poor responding communities. It is also of interest that in this example of poor coverage in the treatment naïve community, the recovery rate of skin mf count 364 days after IVM treatment was only 41.6% of pre-treatment mf count, in contrast to the situation seen in the poor responder communities where the recovery rates were all well in excess of 100% of pre-treatment mf counts.

The evidence from the initial and follow up studies enable a description of the non response or resistance phenotype to multiple treatment with IVM as follows: a) the microfilaricidal response is normal, b) there is early skin microfilarial repopulation, high microfilarial recovery rates one year post ivermectin treatment and many subjects with more than 10 mf/snip, and c) many live microfilariae are present *in utero* in female worms recovered 90 days after treatment. The discovery of such a phenotype should lead to a reevaluation of control strategies in order to prevent the spread of the phenomenon, and a search for the genotypic correlate that would unequivocally confirm the development of resistance.

**Figure 2:**
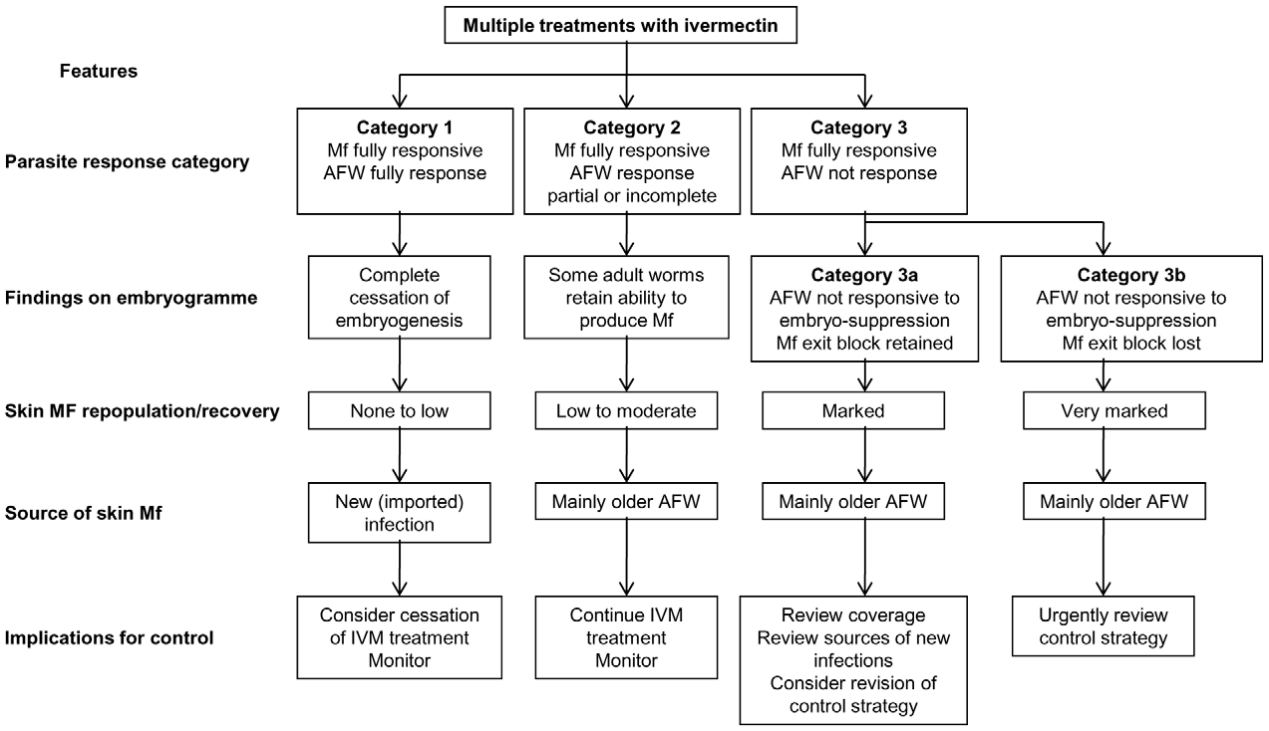
Response profiles of microfilaria and female *O. volvulus* to repeated exposure to ivermectin.

From our studies and evidence from other studies [13], [14], [34], we propose differing patterns of adult female *O. volvulus* responses to repeated rounds of ivermectin treatment. The three response patterns observed are: fully responsive, partial or incomplete response, and non-responsive. The fully responsive is manifested as female worms showing complete cessation of embryogenesis leading to amicrofilaridermia for prolonged periods; any microfilariae detected in a subject harboring only such parasites must originate from an external source (e.g. migration of infected individuals into the community). These parasites fall into “category 1”. The second response pattern is the partial or incomplete response. This involves incomplete cessation of embryogenesis in some female worms, resulting in intermittent low level microfilariadermias beginning six or more months after IVM treatment. These parasites fall into “category 2”. The third response pattern, non-responders, shows minimal interruption in embryogenesis with active intra-uterine mf production associated with rapid repopulation of skin and high recovery rates (“category 3” responders). Of these, a subgroup (category 3a in Figure 2) retained the ability to sequestrate mf in utero. Because of this effect, skin mf do not rise precipitously and abnormal levels of repopulation are only detected three or more months after treatment. The embryogramme shows high levels of microfilarial production associated with the accumulation of a high proportion of degenerate intrauterine mf. In category 3b, a high level of mf production is associated with a low proportion of degenerate intra-uterine mf because the block to their release has been lost and the ivermectin effect is reduced to being only a microfilaricide. There are massive increases in the skin mf soon after one month post-treatment (Awadzi, unpublished). Category 3 responses are likely to allow for parasite transmission for much of the year following ivermectin treatment. It is possible that all of these patterns of response may occur in different communities and the community response category is determined by the proportional distribution of the various adult worm response patterns. The proposed patterns of *O. volvulus* response have been summarized in Figure 2. It is likely that the mechanism of microfilaricidal action of IVM is distinct and separable from the inhibition of the release of mf from the uterus and the suppressive effects of ivermectin on the adult female worm.

At the moment the prevalence of functional blindness, as described by Kennedy and other [30], is almost absent in the study communities, except for a few blind older members of the communities. However, since the risk of developing ocular lesions and blindness is directly related to the intensity of infection [35], [36] it is important to establish strict monitoring of onchocerciasis pathology. Unfortunately, due to a lack of monitoring, the extent of the non response of the adult female worms and the influence it may have on the control of onchocerciasis in areas subjected to annual treatments are at present unknown.

## Supporting Information

Checklist S1 STROBE checklist.
(0.08 MB DOC)

## References

[1] Duke BO, Zea-Flores G, Munoz B (1991). The embryogenesis of Onchocerca volvulus over the first year after a single dose of ivermectin.. Trop Med Parasitol.

[2] World Health Organization (1995). Onchocerciasis and its control: Report of a WHO Expert Committee on Onchocerciasis Control. Technical Report Series No. 852..

[3] Kläger S, Whitworth JA, Post RJ, Chavasse DC, Downham MD (1993). How long do the effects of ivermectin on adult Onchocerca volvulus persist?. Trop Med Parasitol.

[4] Duke BOL, Zea-Flores G, Castro J, Cupp EW, Munoz B (1990). Effect of multiple monthly doses of ivermectin on adult *Onchocerca volvulus*.. Am J Trop Med Hyg.

[5] Duke BO, Pacqué MC, Muñoz B, Greene BM, Taylor HR (1991). Viability of adult *Onchocerca volvulus* after six 2-weekly doses of ivermectin.. Bull World Health Organ.

[6] Duke BO, Zea-Flores G, Castro J, Cupp EW, Munoz B (1992). Effects of three-month doses of ivermectin on adult *Onchocerca volvulus*.. Am J Trop Med Hyg.

[7] Chavasse DC, Post RJ, Davies JB, Whitworth JA (1993). Absence of sperm from the seminal receptacle of female *Onchocerca volvulus* following multiple doses of ivermectin.. Trop Med Parasitol.

[8] Gardon J, Boussinesq M, Kamgno J, Gardon-Wendel N, Demanga-Ngangue (2002). Effects of standard and high doses of ivermectin on adult worms of *Onchocerca volvulus*: a randomised controlled trial.. Lancet.

[9] Plaisier AP, Alley ES, Boatin BA, van Oortmarssen GJ, Remme H (1995). Irreversible effects of ivermectin on adult parasites in onchocerciasis patients in the onchocerciasis control program in west-Africa.. J Inf Dis.

[10] Kläger SL, Whitworth JAG, Downham MD (1996). Viability and fertility of adult *Onchocerca volvulus* after 6 years of treatment with ivermectin.. Trop Med Int Health.

[11] Chavasse DC, Post RJ, Lemoh PA, Whitworth JA (1992). The effect of repeated doses of ivermectin on adult female *Onchocerca volvulus* in Sierra Leone.. Trop Med Parasitol.

[12] Bottomley C, Isham V, Collins RC, Basáñez M-G (2008). Rates of microfilarial production by *Onchocerca volvulus* are not cumulatively reduced by multiple ivermectin treatments.. Parasitology.

[13] Awadzi K, Boakye DA, Edwards G, Opoku NO, Attah SK (2004a). An investigation of persistent microfilaridermias despite multiple treatments with ivermectin, in two onchocerciasis-endemic foci in Ghana.. Ann Trop Med Parasitol.

[14] Awadzi K, Attah SK, Addy ET, Opoku NO, Quartey BT (2004b). Thirty-month follow-up of sub-optimal responders to multiple treatments with ivermectin, in two onchocerciasis-endemic foci in Ghana.. Ann Trop Med Parasitol.

[15] Osei-Atweneboana MY, Eng JK, Boakye DA, Gyapong JO, Prichard RK (2007). Prevalence and intensity of *Onchocerca volvulus* infection and efficacy of ivermectin in endemic communities in Ghana: a two-phase epidemiological study.. Lancet.

[16] Anziani OS, Suarez V, Guglielmone AA, Grande H, Coles GC (2004). Resistance to benzimidazole and macrocyclic lactone anthelmintics in cattle nematodes in Argentina.. Vet Parasitol.

[17] Kotze AC, Dobson RJ, Tyrrell KL, Stein PA (2002). High-level ivermectin resistance in a field isolate of *Haemonchus contortus* associated with a low level of resistance in the larval stage: implications for resistance detection.. Vet Parasitol.

[18] Edward CL, Hoffmann AA (2008). Ivermectin resistance in a horse in Australia.. Vet Rec.

[19] Prichard RK (2001). Genetic variability following selection of *Haemonchus contortus* with anthelmintics.. Trends Parasitol.

[20] Alleman MM, Twum-Danso NA, Thylefors BI (2006). The Mectizan Donation Program - highlights from 2005.. Filaria J.

[21] Eng JK, Prichard RK (2005). A comparison of genetic polymorphism in populations of *Onchocerca volvulus* from untreated- and ivermectin-treated patients.. Mol Biochem Parasitol.

[22] Bourguinat C, Pion SD, Kamgno J, Gardon J, Duke BO (2007). Genetic selection of low fertile *Onchocerca volvulus* by ivermectin treatment.. PLoS Negl Trop Dis.

[23] Ardelli BF, Guerriero SB, Prichard RK (2005). Genomic organization and effects of ivermectin selection on *Onchocerca volvulus* P-glycoprotein.. Mol Biochem Parasitol.

[24] Eng JK, Blackhall WJ, Osei-Atweneboana MY, Bourguinat C, Galazzo D (2006). Ivermectin selection on beta-tubulin: evidence in *Onchocerca volvulus* and *Haemonchus contortus*.. Mol Biochem Parasitol.

[25] Specht S, Brattig N, Büttner M, Büttner DW (2009). Criteria for the differentiation between young and old *Onchocerca volvulus* filariae.. Parasitol Res.

[26] Schulz-Key H (1988). The collagenase technique: how to isolate and examine adult *Onchocerca volvulus* for the evaluation of drug trials.. Trop Med Parasitol.

[27] Schulz-Key H, Albiez EJ, Büttner DW (1977). Isolation of living adult *Onchocerca volvulus* from nodules.. Tropenmed Parasitol.

[28] Schulz-Key H, Jean B, Albiez EJ (1980). Investigations on female *Onchocerca volvulus* for the evaluation of drug trials.. Tropenmed Parasitol.

[29] Alley ES, Plaisier AP, Boatin BA, Dadzie KY, Remme J (1994). The impact of five years of annual ivermectin treatment on skin microfilarial loads in the onchocerciasis focus of Asubende, Ghana.. Trans R Soc Trop Med Hyg.

[30] Kennedy MH, Bertocchi I, Hopkins AD, Meredith SE (2002). The effect of 5 years of annual treatment with ivermectin (Mectizan) on the prevalence and morbidity of onchocerciasis in the village of Gami in the Central African Republic.. Ann Trop Med Parasitol.

[31] Churcher TS, Pion SDS, Osei-Atweneboana MY, Prichard RK, Awadzi K (2009). Identifying sub-optimal responses to ivermectin in the treatment of River Blindness.. Proc Natl Acad Sci, USA.

[32] African Programme for Onchocerciasis Control (2008). http://www.who.int/apoc/about/structure/tcc/Final_Report_TCC_26_English_23_05_08.pdf.

[33] Remme JHF, Amazigo U, Engels D, Barryson A, Yameogo L (2007). Efficacy of ivermectin against *Onchocerca volvulus* in Ghana.. Lancet.

[34] Ali MMM, Mukhtar MM, Baraka OZ, Homeida MMA, Kheir MM (2002). Immunocompetence may be important in the effectiveness of Mectizan (ivermectin) in the treatment of human onchocerciasis.. Acta Trop.

[35] Thylefors B, Brinkmann UK (1977). The microfilarial load in the anterior segment of the eye. A parameter of the intensity of infection of onchocerciasis.. Bull of the World Health Org.

[36] Remme J, Dadzie KY, Rolland A, Thylefors B (1989). Ocular onchocerciasis and intensity of infection in the community. I. West African savanna.. Trop Med Parasitol.

